# Mediators of longitudinal associations between television viewing and eating behaviours in adolescents

**DOI:** 10.1186/1479-5868-8-23

**Published:** 2011-03-30

**Authors:** Natalie Pearson, Kylie Ball, David Crawford

**Affiliations:** 1 Centre for Physical Activity and Nutrition Research, School of Exercise and Nutrition Sciences, Deakin University, 221 Burwood Highway, Victoria, 3125, Australia; 2 School of Sport, Exercise & Health Sciences, Loughborough University, Epinal Way, Loughborough, LE11 3TU, UK

## Abstract

**Background:**

Television viewing has been associated with poor eating behaviours in adolescents. Changing unhealthy eating behaviours is most likely to be achieved by identifying and targeting factors shown to mediate the association between these behaviours. However, little is known about the mediators of the associations between television viewing and eating behaviours. The aim of this study was to examine mediators of the longitudinal associations between television viewing (TV) and eating behaviours among Australian adolescents.

**Method:**

Eating behaviours were assessed using a web-based survey completed by a community-based sample of 1729 adolescents from years 7 and 9 of secondary schools in Victoria, Australia, at baseline (2004-2005) and two years later. TV viewing and the potential mediators (snacking while watching TV and perceived value of TV viewing) were assessed via the web-based survey at baseline.

**Results:**

Adolescents who watched more than two hours of TV/day had higher intakes of energy-dense snacks and beverages, and lower intakes of fruit two years later. Furthermore, the associations between TV viewing and consumption of energy-dense snacks, energy-dense drinks and fruit were mediated by snacking while watching TV. Perceived value of TV viewing mediated the association between TV viewing and consumption of energy-dense snacks, beverages and fruit.

**Conclusion:**

Snacking while watching TV and perceived value of TV viewing mediated the longitudinal association between TV viewing and eating behaviours among adolescents. The efficacy of methods to reduce TV viewing, change snacking habits while watching TV, and address the values that adolescents place on TV viewing should be examined in an effort to promote healthy eating among adolescents.

## Background

Consumption of energy-dense foods and beverages during youth have both immediate and long-term health consequences, including higher body mass index (BMI) [1]. On the other hand, diets rich in fruits and vegetables have important health-protective effects including protection against the development of certain cancers at puberty [2] and in adulthood [3]. Despite this, many young people do not meet current recommendations for fruit and vegetable consumption [4,5], and many regularly consume foods high in fats, sodium and sugar [5,6]. The tendency for eating behaviours established in adolescence to persist into adulthood [7,8] has important implications for nutrition promotion, highlighting the need for a greater understanding of the influences on adolescent eating behaviours.

The television is ubiquitous in most developed countries, and evidence suggests that many young people exceed recommendations for television viewing [9-11]. During the time spent watching television, adolescents expend little energy [12], and are exposed to numerous advertisements that can influence the type of food desired, requested and consumed [13]. Furthermore, television viewing behaviours may act as a complement to stimulate eating by potentially causing a distraction resulting in a lack of awareness of actual food consumption or overlooking food cues, which may lead to overconsumption and increased energy intake [14]. Early research suggests that young people may associate television viewing with eating from a young age, if for example, parents place their children in front of the television with a snack or a meal while they do other household chores [15]. Cross-sectional studies have found that television viewing is associated with lower fruit and vegetable intake [16,17], higher energy-dense beverage consumption [18,19], and higher energy-dense snack consumption [20,21] in adolescents. Research examining longitudinal associations between television viewing and eating among adolescents has shown that television viewing is predictive of lower fruit and calcium intakes and higher sugar-sweetened beverage and fast food intakes after five years [22-24]. While such research begins to explore the television-diet paradigm of adolescents, the extent to which specific mechanism(s) are driving the association have not been investigated [22]. The tendency for television viewing and eating behaviours to aggregate has important implications for nutrition promotion, highlighting the need for a greater understanding of the mechanisms (mediators) underpinning the association between television viewing and eating behaviours. Mediators have been described as processes that intervene between input and output [25]. Understanding the mediators of the associations between television viewing and eating behaviours may help in developing nutrition promotion interventions targeting, for example, high television viewers. However, little is known about the mediators of the associations between television viewing and eating behaviours among adolescents.

A potential explanation for the association between television viewing and eating behaviours among adolescents can be inferred from the existing literature on the determinants of dietary behaviour. There is evidence that snacking while watching television is associated with a higher consumption of energy-dense foods and drinks, and a lower consumption of fruit, vegetables and milk [13,26], and that snacking while watching television differs according to time spent watching television [27-29]. Researchers have proposed that foods eaten in front of the television may be a primary underlying mechanism driving the relationship between television viewing and eating behaviours [30,31]. A further potential explanation can be inferred from behavioural choice theory (BCT). BCT draws on principles of behavioural economics [32] in which the decisions to be active or sedentary (or eat certain foods) are based in part on differences in their accessibility, and on the motivation to engage in the activities, which can be conceptualised as the reinforcing value of the activities [33]. Television viewing has a high reinforcing value for many young people, and is associated with the choice to spend more time watching television [33]. Moreover, television has become the most effective communication medium and as young people spend a large amount of time viewing television it has become an important contributor to their education and socialisation [34,35], which increases it's reinforcing value. It is plausible that the reinforcing value of television could be associated with eating behaviours. For example, studies have shown that the reinforcing value of television is associated with increased frequency of children's attempts to influence the purchasing of certain foods at the supermarket [34], and with increased preferences for foods viewed on television [36].

To our knowledge, no previous studies have examined whether snacking while watching television, or adolescents' perceived value of television viewing mediate the prospective associations between television viewing and eating behaviours among adolescents. This study aimed to (i) examine the associations between time spent watching television among adolescents at baseline (T1) and eating behaviours two-years later (T2), and (ii) to examine whether associations between television viewing at T1 and eating behaviours at T2 are mediated by frequency of snacking while watching TV, and adolescents' perceived value of television viewing.

## Methods

### Study procedure

The Youth Eating Patterns (YEP) study is a longitudinal study of dietary habits among adolescents in Melbourne, Victoria, Australia. All co-educational state (government) and Catholic secondary schools (years 7 to 12) with enrolments over 200, located in the southern metropolitan region of Melbourne and the non-metropolitan region of Gippsland (Victoria), to the east of Melbourne, were invited to participate in the study. Of the 70 schools (47 metropolitan and 23 non-metropolitan) that met these criteria, 37 schools (20 metropolitan and 17 non-metropolitan) agreed to participate. The response rate of schools agreeing to participate in the survey was greater in non-metropolitan Victoria (73.9%) compared with Metropolitan Melbourne (42.5%). The YEP survey is an online food habits survey and was administered by teachers during a class when students had access to computers. The survey was administered during 2004-2005 (baseline, T1), and again two years later in 2006-2007 (follow-up, T2) using identical procedures. Study procedures were approved by the Ethics committee of Deakin University, the Victorian Department of Education and Training, and the Catholic Education Office. YEP survey participant recruitment and study procedures has been provided in previous publications [37,38].

### Subjects

All students (n = 9,842) from year 7 (aged 12-13 years) and year 9 (aged 14-15 years) from participating schools were invited to complete the online survey at baseline. Teachers distributed parental consent forms via students asking permission for their child to participate in the study. Parental consent was obtained for 4,502 (46%) of all eligible students. Online surveys were completed at baseline by 3,264 adolescents who provided assent and were present on the day of data collection. Of these, 1,884 (58%) completed the YEP survey at the 2-year follow-up.

The present analyses are based on the subset of 1729 adolescents who had non-missing data for all of the variables examined in this study (i.e. T1 TV viewing, T1 potential mediators, and T2 dietary outcomes). Comparison of these 1729 adolescents with those who were not followed up (n = 1,380) showed no significant differences in consumption of energy-dense snacks, energy-dense drinks, and fruit. However, a significantly (p < 0.05) higher proportion of adolescents that were followed up, compared to those who were not, were girls (55.4% compared to 44.6%), and were in year 7 at baseline (65.2% compared to 38.4%).

### Measures

Demographic characteristics of adolescents including date of birth, school year and gender were collected at T1.

### Adolescent eating behaviours

Consistent with other large-scale studies of dietary intake and eating behaviours of adolescents [39], food intake was assessed at baseline (T1) and follow-up (T2) using a food frequency questionnaire (FFQ). This FFQ was based on previously validated indices of food intake [40] and is described in detail in previous publications [37,38]. Respondents indicated how frequently they had consumed 37 food items during the previous month. Seven responses categories ranged from 'never or not in the last month' to 'several times a day'.

The present analyses are based on a subset of nine food items from the FFQ, which were categorised into three food groups: energy-dense snacks, energy-dense drinks and fruit. These foods were selected due to their importance in contributing to the healthfulness of overall diet. The frequency of consumption of the nine food items in the past month was converted to a daily equivalent, which is an established method [41] that has been used in other dietary studies [39,42]. Daily equivalent scores were calculated as follows: not in the last month (0.00 per day), several times per month (0.11 per day), once a week (0.14 per day), a few times a week (0.36 per day), on most days (0.71 per day), once per day (1.00 per day) and several times per day (2.50 per day). The daily intake for each of the three food groups was calculated by summing the daily equivalent scores for the food items in each food group. The estimated daily intake of the energy-dense snack group included the summed equivalents of four items (confectionary, cakes, sweet biscuits and potato crisps/salty snacks). The estimated daily intake of the energy-dense drink group included the summed equivalents of four items (non-diet cordial, non-diet soft-drinks, energy-drinks and sports drinks). The daily intake of fruit included fruit as one item (fresh, canned, frozen or dried).

### Adolescent television viewing

Adolescents reported how much time (hours/minutes) they usually spend watching television/DVDs/movies in a typical week (Monday to Friday) and on a typical weekend (Saturday and Sunday). To compute the average, daily minutes spent watching television/DVDs/movies during the week and on the weekend were summed and divided by seven. Consistent with recommendations for screen time in youth [9], T1 television viewing was dichotomised as ≤2 hours/day and >2 hours/day for the present analyses. Throughout the manuscript, this variable is referred to as television (TV) viewing, although it also included DVD/movie watching.

### Mediators

Eating snacks while watching television was assessed at T1 via one item 'over the past month, how often have you snacked while watching television?' Five response categories were 'not in the last month', 'once or twice a month', 'once or twice a week', 'most days', and 'everyday'.

The liking of/beliefs about television viewing or the 'value' that adolescents placed on watching television was assessed with three items at T1: the best way to relax is to watch television, if I don't watch television I think my day is incomplete, and for me television is the best form of entertainment. Responses for the three items were provided on a three-point Likert scale: 'disagree', 'not sure', and 'agree' and were summed (Cronbach's alpha = 0.71).

In experimental research, the reinforcing value of sedentary behaviour is assessed using computer games in a lab setting [43,44]. This measure was not feasible in our study and thus we have used a measure of liking and beliefs/values since these have been shown to be related to reinforcing value. For example Robinson and Berridge [45] suggest that liking may be an important characteristic of many things that people find reinforcing, and repeated exposure to something that is liked will lead to it becoming reinforcing. Throughout the manuscript authors refer to the measure used here as 'perceived value of television viewing'.

### Statistical analysis

Initial analyses were conducted using the SPSS statistical software package version 17.0 (SPSS Inc., Chicago, IL, USA). Descriptive statistics were used to summarise the demographics, potential mediators, television viewing, and eating characteristics of the sample. All further analyses were conducted using Stata 11 (Stata Corp, College Station TX, 2003).

Firstly, linear regression analyses were used to examine associations between TV viewing and the two proposed mediators, and between the proposed mediators and adolescent eating behaviours. Secondly, as suggested by Cerin et al. [46] the mediating effects of eating snacks while watching television, and the value of television viewing on the association between T1 television viewing and T2 eating behaviours were assessed using the Freedman-Schatzkin test of mediation [47]. The Freedman-Schatzkin test is based on the difference in the unstandardised regression coefficient for the association between an independent (T1 television viewing) and dependent variable (T2 eating behaviour), unadjusted (τ) and adjusted (τ') for the proposed mediator(s). The significance of the mediating effect is computed by dividing this difference (τ-τ') by its standard error and comparing the obtained value to a t-distribution with n-2 degrees of freedom. In this study, single mediator models were tested. All regression models were adjusted for gender and age (year level), and accounted for potential clustering by school (unit of recruitment) using the 'cluster' command.

## Results

The majority of the adolescent sample at baseline were girls (55%) and in year 7 (65%). The mean age of adolescents was 13.2 years (SD 1.6). Table 1 shows the distribution of demographic characteristics, TV viewing, potential mediators, and dietary outcomes at T1 and T2 according to adolescent gender and year level. There were some significant differences by gender and year level. Males placed a higher value on TV viewing compared to females; males had higher consumptions of energy-dense drinks at T1 and T2, whereas females had higher consumptions of fruit at T1 and T2. A higher percentage of adolescents in Year 7 had mothers with lower education levels compared to those in Year 9. A higher percentage of adolescents in Year 9 watched TV for more than 2 hours/day compared to those in Year 7. Adolescents in Year 9 reported snacking while watching TV more frequently than those in Year 7; adolescents in Year 7 placed a higher value on TV viewing than those in Year 9.

**Table 1:** Description of socio-demographic characteristics, television viewing, potential mediators, and eating behaviours.

	Gender		Year level	
	
	Male (n = 774)	Female (n = 955)	Year 7 (n = 1107)	Year 9 (n = 622)
**Maternal education (%)**				
Low	340 (44)	430 (45)	520 (47)	249 (40)***
Medium	232 (30)	306 (32)	332 (30)	211 (34)
High	202 (26)	219 (23)	255 (23)	162 (26)
**School region (n, %)**				
Metropolitan	565 (73)	688 (72)	797 (72)	460 (74)
Rural	209 (27)	267 (28)	310 (28)	162 (26)
**TV viewing (n, %)**				
≤2 hours/day	382 (49)	517 (54)	603 (54)	296 (48)
>2 hours/day	392 (51)	438 (46)	504 (46)	326 (52)**
**T1 potential mediators**				
Snacked while watching the TV (mean, SD; range 1-5)	3.46 (1.14)	3.45 (1.06)	3.42 (1.21)	3.52 (1.04)*
Value of TV viewing (mean, SD; range 3-9)	5.39 (1.91)	4.98 (1.85)***	5.23 (1.87)	5.04 (1.90)*
**T1 dietary behaviours (mean serves/day, SD)**				
Energy-dense snack foods	1.27 (1.56)	1.19 (1.32)	1.23 (1.42)	1.23 (1.46)
Energy-dense drinks	1.24 (1.49)	0.78 (1.05)***	1.03 (1.32)	0.91 (1.23)
Fruit	0.84 (0.84)	0.98 (0.85)***	0.94 (0.86)	0.87 (0.83)
**T2 dietary behaviours (mean serves/day, SD)**				
Energy-dense snack foods	1.11 (1.34)	1.10 (1.12)	1.08 (1.28)	0.97 (1.12)
Energy-dense drinks	1.13 (1.55)	0.63 (1.04)	0.88 (1.37)	0.81 (1.20)
Fruit	0.84 (0.83)	1.10 (0.90)	1.01 (0.88)	0.93 (0.88)

Chi-square test (TV viewing, maternal education and school region) or univariate ANOVAs.

*p < 0.05, **p < 0.01, ***p < 0.001

T1: baseline assessments took place in 2004-2005. T2: Follow-up assessments took place in 2006-2007.

Linear regression analyses, adjusted for adolescent gender and year level, revealed that TV viewing was positively associated with snacking while watching TV (β = 0.01, 95% CI 0.005-0.006, p < 0.001) and with perceived value of TV viewing (β = 0.01, 95% CI 0.008-0.01, p < 0.001). Further linear regression analyses revealed that snacking while watching TV and perceived value of TV viewing were positively associated with energy-dense snack and energy-dense drink consumption, and negatively associated with fruit consumption (Table 2).

**Table 2:** Associations between potential mediating variables and adolescent eating behaviours at T2 (outcome variables)

	T2 Energy-dense snack consumption		T2 Energy-dense drink consumption		T2 Fruit consumption	
			
	Unstandardised regression coefficient (95% CI)	P-value	Unstandardised regression coefficient (95% CI)	P-value	Unstandardised regression coefficient (95% CI)	P-value
**Potential mediating variables**						
Eating snacks while watching TV	0.26 (0.21-0.31)	<0.001	0.14 (0.09-0.20)	<0.001	-0.06 (-0.10--0.03)	0.001
Value of TV viewing	0.10 (0.07-0.13)	<0.001	0.11 (0.07-0.15)	<0.001	-0.05 (-0.07--0.03)	<0.001

Linear regression analyses adjusted for gender and year level, and accounting for potential clustering by school (unit of recruitment) using the 'cluster' command.

Tables 3, 4 and 5 show the results of the mediation analyses procedures applied to the prediction of T2 energy-dense snacks, energy-dense drinks, and fruit consumption, respectively. Prediction of energy-dense snack consumption by TV viewing declined significantly after controlling for both mediators, suggesting that variations in energy-dense snack consumption by TV viewing were mediated by these constructs (Table 3). Based on the magnitude of the t-values, eating snacks while watching TV played the greatest mediating role (*t*(1727) = 8.96, p < 0.001).

**Table 3:** Analyses of potential mediators of the association between TV viewing (T1) and ED snack consumption (T2).

	τ'	SE	τ - τ'	SE	*t*	*R^2^*	*P*-value
**τ **(mediator-unadjusted coefficient regressing ED snack consumption at T2 on TV viewing at T1) = 0.27 (SE = 0.06)***							
**Potential mediators**							
Eats snacks while watching TV	0.14**	0.06	0.13	0.03	8.96	0.06	<0.001
Value of TV viewing	0.17**	0.05	0.10	0.04	5.14	0.03	<0.001

τ, unstandardised regression coefficient for association between TV viewing and ED snack consumption, adjusting for confounders (gender and year level) and accounting for potential clustering by school (unit of recruitment) using the 'cluster' command, before adjustment for mediator; τ', unstandardised regression coefficient for association between TV viewing and ED snack consumption, adjusting for confounders (gender and year level), and mediator; τ- τ', difference between the two regression coefficients, which when divided by its standard error, can be compared against a *t*-distribution with *n *- 2 degrees of freedom; SE, standard error; ns, not significant.

p < 0.05, **p < 0.01, ***p < 0.001

**Table 4:** Analyses of potential mediators of the association between TV viewing (T1) and ED drink consumption (T2).

	τ'	SE	τ - τ'	SE	*t*	*R^2^*	*P*-value
**τ **(mediator-unadjusted coefficient regressing ED drink consumption at T2 on TV viewing at T1) = 0.20 (SE = 0.07)**							
**Potential mediators**							
Eats snacks while watching TV	0.14*	0.06	0.07	0.03	4.14	0.05	<0.001
Value of TV viewing	0.07 ns	0.06	0.13	0.04	6.17	0.06	<0.001

τ, unstandardised regression coefficient for association between TV viewing and ED drink consumption, adjusting for confounders (gender and year level) and accounting for potential clustering by school (unit of recruitment) using the 'cluster' command, before adjustment for mediator; τ', unstandardised regression coefficient for association between TV viewing and ED drink consumption, adjusting for confounders (gender and year level), and mediator; τ- τ', difference between the two regression coefficients, which when divided by its standard error, can be compared against a *t*-distribution with *n *- 2 degrees of freedom; SE, standard error; ns, not significant.

*p < 0.05, **p < 0.01, ***p < 0.001

**Table 5:** Analyses of potential mediators of the association between TV viewing (T1) and fruit consumption (T2).

	τ'	SE	τ - τ'	SE	*t*	*R^2^*	*P*-value
**τ **(mediator-unadjusted coefficient regressing fruit consumption at T2 on TV viewing at T1) = -0.17 (SE = 0.04)***							
**Potential mediators**							
Eats snacks while watching TV	-0.14***	0.04	-0.03	0.02	-3.10	0.04	0.002
Value of TV viewing	-0.12**	0.04	-0.05	0.03	-3.85	0.04	<0.001

τ, unstandardised regression coefficient for association between TV viewing and fruit consumption, adjusting for confounders (gender and year level) and accounting for potential clustering by school (unit of recruitment) using the 'cluster' command, before adjustment for mediator; τ', unstandardised regression coefficient for association between TV viewing and fruit consumption, adjusting for confounders (gender and year level), and mediator; τ- τ', difference between the two regression coefficients, which when divided by its standard error, can be compared against a *t*-distribution with *n *- 2 degrees of freedom; SE, standard error; ns, not significant.

*p < 0.05, **p < 0.01, ***p < 0.001

Prediction of energy-dense drink consumption by TV viewing also declined significantly after controlling for both potential mediators, suggesting that variations in energy-dense drink consumption by TV viewing were mediated by these constructs (Table 4). Based on the magnitude of the t-values, the perceived value of TV viewing played the greatest mediating role (*t*(1727) = 6.17, p < 0.001).

Table 5 shows that both snacking while watching television and the perceived value of TV viewing mediated the negative association between TV viewing and fruit consumption, with perceived value of television viewing playing the greatest mediating role (*t*(1727) = -3.85, p < 0.001).

## Discussion

The present study examined the mediating influences of snacking while watching television and the perceived value of television viewing on the longitudinal association between television viewing and eating behaviours in a large regionally diverse sample of Australian adolescents. Adolescents who watched more than two hours of television per day had higher intakes of energy-dense snacks and drinks, and lower intakes of fruit two years later. Furthermore, the associations were mediated by snacking while watching television and perceived value of television viewing. The importance of explicating the mechanisms through which television viewing influences adolescent eating behaviours not only relates to the advancement of scientific knowledge: Understanding these mechanisms is also crucial because it offers potential avenues for targeted intervention programmes.

Cross-sectional studies have found associations between television viewing and unhealthy eating behaviours in pre-school children [48,49], school-aged children [50,51], adolescents [52,53], and adults [54,55]. Longitudinal studies examining the associations between television viewing and eating behaviours are becoming more prevalent; however, data on adolescents comes predominantly from the US. Data from project EAT has shown television viewing to be predictive of lower fruit and calcium intakes, and higher sugar-sweetened beverage and fast food intakes after five years [22-24]. Other prospective research from the US has shown that for each hour increase in television viewing adolescents consumed additional energy intake of 106 kcal a day [56] and decreased intakes of fruit and vegetables [57]. Findings from the current study support and add to previous studies by showing that adolescents in Australia who watch more television are more likely to have poorer eating behaviours. The present study also supports previous literature showing that television viewing is associated with snacking while watching television [28,29], and that snacking while watching television is positively associated with consumption of energy-dense snacks and drinks, and negatively associated with consumption of fruit [13,26]. For some young people a significant proportion of their daily energy intake is consumed while watching television [30]. Experimental studies have shown that watching television while eating may cause a distraction resulting in a delay in normal mealtime satiation and a reduction in internal satiety signals [58,59], which may lead to overeating. Given that adolescence is a critical developmental period, during which lifelong behaviours are formed [60], the present findings suggest that examining methods to modify television viewing and snacking and eating behaviours in front of the television should be a priority for nutrition promotion.

The present study advances previous findings by demonstrating that variations in snacking while watching television play an important role in mediating the associations between television viewing and adolescents' intakes of energy-dense snacks and drinks, and fruit 2-years later. This is a novel finding, since to the best of our knowledge, no previous studies have examined potential mediating influences of the association between television viewing and eating behaviours in adolescents. Snacking while watching television partially explained (mediated) the positive association between television viewing and consumption of energy-dense snacks and drinks, and the negative association between television viewing and consumption of fruit. While watching television, adolescents are exposed to a larger number of advertisements, which are more often for energy-dense foods compared to fresh foods such as fruits and vegetables [61]. Previous research has shown that television advertising directed at young people influences their preferences, requests and short-term consumption of foods and beverage products advertised on television [13]. It may be, therefore, that those adolescents who watch more television, subsequently prefer, request and consume as snacks more of the types of foods advertised, which would explain the associations observed between these variables. Such findings suggest that reducing television viewing and changing snacking habits while watching television may be important strategies for improving the overall healthfulness of adolescent eating behaviours.

The present study found that the perceived value of television viewing varied by the amount of time spent watching television. Adolescents who watched more television placed a higher value on television viewing than those who watched less. This part of the model was cross-sectional so we cannot rule out reverse causality. For many young people, sedentary behaviours such as television viewing have a high reinforcing value (motivation to participate), so they are more likely to become sedentary and less likely to develop a regular physical activity habit [62]. Furthermore, accessibility of sedentary behaviours is much easier than that for physical activity for most people [33], which increases the amount of motivation people need to choose activity over more sedentary pursuits. Experimental research using positive reinforcement to reduce sedentary behaviours and increase the accessibility of physical activity has had mixed success at increasing physical activity [43,63,64]. Future research should examine the applicability of such an approach in real world settings.

Further analyses showed that the value that adolescents placed on television viewing partially explained (mediated) the association between television viewing and consumption of energy-dense snacks, drinks and fruit. Again, this is a novel finding, since to the best of our knowledge, no previous studies have examined potential mediating influences of the association between television viewing and eating behaviours in adolescents. A potential explanation for such associations is that if adolescents place a high value on watching the television, they are more likely to take on board the messages promoted, which as mentioned above, are likely to encourage the consumption of energy-dense foods and drinks rather than more healthy snacks (e.g. fruit). Future research aiming to improve adolescent eating behaviours should assess the efficacy of methods to address the values that adolescents place on television viewing.

In considering these findings it is important to acknowledge the limitations of the study. There was some loss of participants at follow-up and some differences between those with follow-up data and those with baseline only data, although the sample at follow-up remained diverse. All data were collected by self-report and are subject to socially desirable response bias or other misreporting. T2 data is not available for the examined mediating variables, and thus we were not able to examine changes in these variables over time. When using observational data, prospective relationships can, as for cross-sectional data, be due to a third antecedent. Thus, we do not assume causality. Strengths of the study include its longitudinal design, the large regionally diverse sample, and the use of powerful statistical mediation techniques.

## Conclusion

Acknowledging these limitations, the present results are important since little is known about the mechanisms underlying the associations between television viewing and eating behaviours among adolescents. The findings suggest that future research should assess the efficacy of methods to reduce television viewing, change snacking habits while watching television, and address the values that adolescents place on television viewing in an effort to promote healthy eating among adolescents.

## Competing interests

The authors declare that they have no competing interests.

## Authors' contributions

NP carried out the statistical analyses and drafted the paper. KB was involved in the design and conduct of the Youth Eating Patterns (YEP) study and contributed to the drafting of the paper. DC was involved in the design and conduct of the Youth Eating Patterns (YEP) study and contributed to the drafting of the paper. All authors read and approved the final manuscript.
